# Student’s Manual of Venereal Diseases

**Published:** 1886-08

**Authors:** 


### BOOK REVIEWS.

1. THE STUDENT'S MANUAL OF VENEREAL DISEASES; *being a Concise Description of Those Affections and of Their Treatment. By* BERKELEY HILL, M. D., *Professor of Clinical Surgery in University College, London, etc.; and* ARTHUR COOPER, M. D., *Surgeon to the Westminster General Dispensary, etc. Fourth edition, revised. Philadelphia:* P. BLAKISTON, SON & CO. *1886.*

